# Severe TSH Elevation and Pituitary Enlargement After Changing Thyroid Replacement to Compounded T4/T3 Therapy

**DOI:** 10.1177/2324709616661834

**Published:** 2016-08-02

**Authors:** Adlai L. Pappy, Nelson Oyesiku, Adriana Ioachimescu

**Affiliations:** 1 Department of Medicine and Neurosurgery, Emory University, Atlanta, GA, USA

**Keywords:** hypothyroidism, compounded T4/T3, levothyroxine, pituitary hyperplasia, pituitary enlargement

## Abstract

We present the first case of iatrogenic hypothyroidism as a result of compounded thyroid hormone (T4/T3) therapy. The thyroid replacement was changed from 175 µg levothyroxine (LT4) to 57/13.5 µg compounded T4/T3 daily in order to improve the T3 level, despite normal thyroid-stimulating hormone (TSH). This resulted in clinical manifestations of hypothyroidism and high TSH level (150 µIU/mL). Six months later, the patient was referred to our clinic for abnormal pituitary magnetic resonance imaging. On reinitiating a physiologic dose of LT4, clinical and biochemical abnormalities resolved and the pituitary gland size decreased. Our case emphasizes the importance of using TSH level to gauge dose adjustments in primary hypothyroidism. Also, it underscores the current American Thyroid Association recommendation against routine use of compounded thyroid hormone therapy.

## Introduction

Most patients with hypothyroidism experience resolution of symptoms once thyroid-stimulating hormone (TSH) normalized with levothyroxine (LT4) replacement. However, some thyroidectomized patients replaced with LT4 have low circulating T3 despite normal TSH levels.^1-4^ Studies that assessed desiccated thyroid extracts (DTE) in patients dissatisfied with LT4 have shown some patients prefer DTE.^5^ Both DTE and compounded T4/T3 have been used in patients who desire T3 along with T4 replacement. Published cases on adverse effects of compounded thyroid preparations are limited to thyrotoxicosis.^6,7^ We present a unique case showing how inappropriate compounding T4/T3 treatment can cause iatrogenic hypothyroidism, severely elevated TSH, elevated creatine kinase (CK), and pituitary enlargement.

## Case

**Figure 1.**
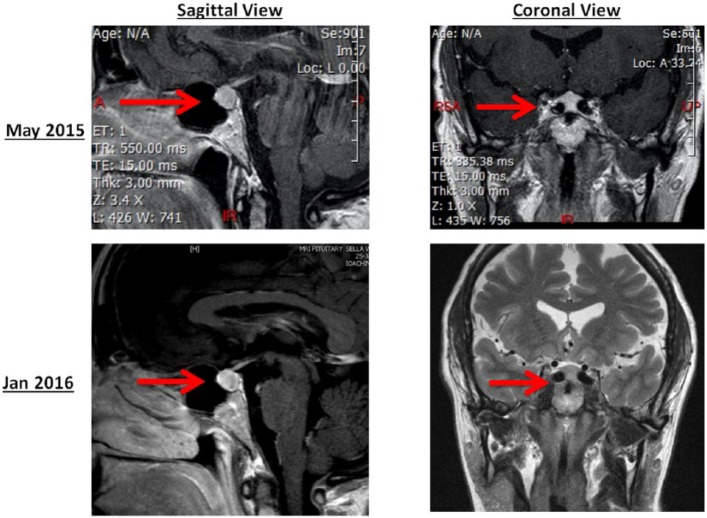
MRI of the pituitary gland at presentation (May 2015) and after restoring euthyroidism (Jan 2016).

A 63-year-old woman was seen in our clinic in July 2015 for abnormal pituitary magnetic resonance imaging (MRI) and elevated TSH of 150 µU/mL. Past medical history was only remarkable for autoimmune hypothyroidism diagnosed in 1981. She had been taking LT4 175 µg/day, which yielded normal thyroid tests. However, in December 2014, she was switched to sustained-release compounded synthetic T4/T3 therapy 57/13.5 µg/day in order to improve T3 level. In February 2015, she began experiencing fatigue, bloating, and constipation. In April 2015, TSH was 148 µIU/mL (normal level [nl]: 0.4-4.5; Table 1). She became increasingly tired, gained weight, and developed headaches, muscle cramps, and insomnia. In May 2015, CK was 400 U/L (nl: 26-140) and MRI was read as pituitary enlargement and a 5 mm hypodense lesion (Figure 1). At this time, the compounded T4/T3 dose thyroid was increased from 57/13.5 µg to 66.5/15.75 µg. In June 2015, TSH remained >100 µIU/L, and the dose was further increased to 76/18 µg daily.

**Table 1.** Laboratory Data and Thyroid Hormone Replacement Dose.

Date	TSH	Free T4	Free T3	CK	Thyroid Therapy Dose
	.40-4.50 (µlU/ml)	0.82-1.77 (ng/dl)	2.00-4.40 (pg/ml)	21-215 (IU/L)	
Sep 2012	3.00	1.3	2.7		LT4 112 µg
Jun 2013	7.20	1.7	2.8		LT4 125 µg
Nov 2013	2.03	1.8	2.8		LT4 150 µg
May 2014	0.10	2.3	2.7		LT4 150 µg
Dec 2014	0.60	1.7	2.3		LT4 175 µg
Apr 2015	148.30		0.5		T4 57/T3 13.5 µg
Apr 2015	150.80		0.9		T4 57/T3 13.5 µg
Jun 2015	>100.00	0.3	1.5	400	T4 63.3/T3 15 µg
Jul 2015	147.00		0.8		T4 76/T3 18 µg
Jul 2015					T4 137 µg
Aug 2015	8.46	2.0		248	T4 137 µg
Sep 2015	2.63			143	T4 137 µg

Abbreviations: TSH, thyroid-stimulating hormone; CK, creatine kinase.

Family history was remarkable for hypothyroidism in patient’s mother and sister. Medications were compounded T4/T3 sustained release 76 µg/18 µg once daily in the morning 1 hour before breakfast, and a compounded cream mixture of estrogen, progesterone, testosterone, and DHEA. Review of symptoms was positive for fatigue, headaches, difficulty sleeping, vision changes, and constipation. The pertinent positives on the physical exam were generalized obesity with a body mass index of 32.9 kg/m^2^, blood pressure at 146/85 mm Hg, and dry skin. The pertinent negatives were normal visual fields on confrontation, normal abdomen, normal gait, normal thyroid exam, and strength with a full range of motion. Additional endocrine labs were normal: prolactin 19.2 ng/mL, morning cortisol 13 µg/dL, ACTH 41 pg/mL, and insulin-like growth factor 92 ng/mL.

Our diagnosis was iatrogenic hypothyroidism due to the change of LT4 to compounded T4/T3. After review of doses of thyroid replacement taken over time (Table 1), the patient was reinitiated on LT4 137 µg/day, which also was concordant with the 1.6 µg/kg weight-based dose. The pituitary enlargement was hypothesized to be most likely due to pituitary hyperplasia rather than a pituitary adenoma. Elevated CK was attributed to uncontrolled hypothyroidism. After 3 weeks, laboratory data improved: TSH 8.46 µIU/mL and CK 248 U/L (21-215 U/L). In September 2016, TSH and CK normalized (2.63 µIU/mL and 143 U/L, respectively). During this time, the patient reported symptoms subsided and weight loss of 7 kg. MRI was repeated in January 2016 and showed a decrease in pituitary gland size and no definite focal lesion. The neuroradiologist also noted *medialized or “kissing” internal carotids*.

## Discussion

This case highlights how inappropriate synthetic compounded therapy dosing can cause iatrogenic hypothyroidism, pituitary gland enlargement, and elevated CK. Prior cases indicate compounded thyroid preparations caused severe thyrotoxicosis due to preparation errors.^6,7^ Compounded drugs are not Food and Drug Administration–approved as their safety and effectiveness have not been verified. The Food and Drug Administration warns about possible health risks with compounded drugs that do not meet the federal quality standards.^8^ Furthermore, the American Thyroid Association recommends LT4 alone as the treatment for hypothyroidism and discourages use of compounded T4/T3.^9^

Our patient was converted from LT4 to compounded T4/T3 despite normal TSH levels in an attempt to “make T3 better.” In addition, she developed manifestations of hypothyroidism within a few weeks of switching from LT4 to compounded T4/T3, but TSH was only repeated after 4 months. The American Thyroid Association recommends TSH levels should be the primary determinant in gauging the treatment of primary hypothyroidism.^10^ Guidelines also recommend a follow-up evaluation should be done 4 to 6 weeks after changing the thyroid hormone preparation or the dose.^9,10^

Our literature review identified 13 randomized controlled trials, 3 meta-analyses, and 1 systematic review comparing the efficacy of LT4 to combination T4/T3.^11-23^ Among the trials, 8 found no difference in TSH levels, 3 found significantly higher TSH in the T4/T3 than in the LT4 group, and 1 study showed higher TSH in the LT4 compared with the T4/T3 group. In the Appelhof et al^11^ study, there were 3 different arms that used T4/T3 ratios of 10:1, 5:1, and 1:0. The 10:1 arm showed no difference in TSH levels when compared to LT4 alone; yet, the 5:1 arm showed a significantly lower TSH level compared to LT4.^11^ The 5:1 arm in the Appelhof et al^11^ study was the only one that showed a mean TSH level out of range at 0.07 µU/L (nl: 0.1-4.0) yielded by the T4/T3 therapy. Regarding patient preference for LT4 versus combination T4/T3 therapy, 8 studies^11,13,14,18,20,21,23,24^ showed no difference, while 4 studies^12,17,19,22^ showed preference for combination T4/T3. Most studies indicated an improvement in the quality of life in both treatment groups. None of the 3 meta-analyses showed a significant advantage of the combination T4/T3 over LT4 on mood, health-related quality of life,^25-27^ or cognition.^25,27^ Furthermore, Escobar-Morreale’s systematic review showed that the benefits of combination T4/T3 therapy on psychological outcomes were heterogeneous and not reproducible.^24^

Our patient was converted from a daily dose of 175 µg LT4 to a lower dose of 57/13.5 µg compounded T4/T3 dose. After 2 successive increases in T4/T3 doses, the prescribed dose of T4 was still too low.^8-20^ This prompted our further investigation as to why the physician prescribing the compounded T4/T3 chose these doses. We found a compounding pharmacy publication that recommended a T4/T3 ratio of 11:1 and twice daily T3 administration.^28^ However, when contacted for guidance regarding the conversion from LT4 to compounded T4/T3, 3 compounding pharmacies indicated a ratio based on equivalent doses of T4/T3 with those found in DTE (Supplement 1, available online at http://hic.sagepub.com/supplemental). It appears our patient’s dose was extrapolated from the conversion of a patient taking LT4 alone to DTE. A previous study showed that DTE taken once daily could be a reasonable alternative for a subgroup of hypothyroid patients.^5^ However, none of the 13 clinical trials administered synthetic compounded T4 in doses equivalent to the amount of T4 in a once daily dosing of DTE (Table 2).

**Table 2.** Studies Comparing Combination T4/T3 With LT4 Alone.

Source of Conversion	Combination T4/T3	T4 (Levo)
Pharmacy conversion chart	T4 (Levo) 57/T3 (Lio) 13.5 µg	150 µg
Appelhof et al, 2005	T4 (Levo) 125/ T3 (Lio) 25 µg	150 µg
Appelhof et al, 2005	T4 (Levo) 125/T3 (Lio) 12.5 µg	150 µg
Bunevicious et al, 1999	T4 (Levo) 100/T3 (Lio) 12.5 µg	150 µg
Bunevicious et al, 2002	T4 (Levo) 100/T3 (Lio) 10 µg	150 µg
Clyde et al, 2003	T4 (Levo) 100/T3 (Lio) 15 µg	150 µg
Escobar-Morreale et al, 2005	T4 (Levo) 75/T3 (Lio) 5 µg	T4 100 µg
Fadeyev et al, 2010	T4 (Levo) 125/T3 (Lio) 12.5 µg	T4 1.6 µg/kg/day
Nygaard et al, 2009	T4 (Levo) 100/T3 (Lio) 20 or 50 µg	150 µg
Rodriguez et al, 2005	T4 (Levo) 100/T3 (Lio) 10 µg	150 µg
Saravanan et al, 2005	T4 (Levo) 100/T3 (Lio) 10 µg	150 µg
Sawka et al, 2003	T4 (Levo) 100/T3 (Lio) 25 µg	150 µg
Siegmund et al, 2004	T4 (Levo) 142.5/T3 (Lio) 7.5 µg	150 µg
Valizadeh et al, 2009	T4 (Levo) 100/T3 (Lio) 12.5µg	150 µg
Walsh et al, 2003	T4 (Levo) 100/T3 (Lio) 10 µg	150 µg

Other factors that might have contributed to the subtherapeutic effect of compounded T4/T3 are differences in bioavailability and degradation of the medication delivered as a 90-day supply. Yet onset of hypothyroid symptoms within few weeks of the change in medication is more consistent with using a significantly lower dose than appropriate. Another factor highlighted by reports of thyrotoxicosis as a result of compounded thyroid hormone preparations was that tablets did not contain the listed doses of these medicines.^7^ This was not tested in our case given the inappropriate dose conversion.

Besides biochemically severe iatrogenic hypothyroidism, our patient had other salient features. First, enlargement of the pituitary gland on the MRI was detected on the initial MRI and attributed to a pituitary adenoma. This was at least partially due to TSH cell hyperplasia. In 1851, Niepce showed a correlation between primary hypothyroidism and pituitary gland enlargement.^29^ Further reports showed excessive TSH stimulation by TRH can cause pituitary enlargement due to thyrotroph and lactotroph hyperplasia.^30,31^ This is usually reversible after control of primary hypothyroidism. The MRI also indicated *medialized or “kissing” internal carotids*. This anatomical variant can cause an upward convexity of the pituitary gland that can be misdiagnosed as a pituitary tumor and lead to unnecessary surgery.^32,33^ Finally, our patient had muscle symptoms along with CK elevation, which resolved with correction of hypothyroidism. This is in concordance with previous reports of hypothyroidism presenting with polymyositis-like symptoms causing an elevated CK.^34^

## Conclusion

Iatrogenic hypothyroidism, pituitary gland enlargement, and elevated muscle enzymes can occur with synthetic compounded T4/T3 preparations. The optimum ratio of T4 to T3 has not been determined, and such therapy is not subject to the Food and Drug Administration regulation. Therefore, we agree with the American Thyroid Association and American Association of Clinical Endocrinologists guidelines that hypothyroidism should be treated with LT4. In addition, we emphasize that TSH level should be the main factor to gauge dose adjustments in primary hypothyroidism.

## Supplementary Material

Supplementary material
