# Supplementary material for: Severe TSH Elevation and Pituitary Enlargement After Changing Thyroid Replacement to Compounded T4/T3 Therapy
Source: J Investig Med High Impact Case Rep. 2016 Aug 2;4(3):2324709616661834. doi: 10.1177/2324709616661834 (PMC4973410; doi:10.1177/2324709616661834)
Supplement: Supplementary material [file Supplement_1.pdf]

Bio-Thyroid		Armour Thyroid	Synthroid/ Levothroid/ Levoxyl
(Immediate Release Capsules, Sustained Release Capsules, +/- Conversion Co-Factors)*		(Thyroid Tablets)	(Levothyroxine Tablets)
Compounded		Desiccated, Porcine	Synthetic
T4 (4.2)	T3 (1)	T4/T3	T4
7.6 mcg	1.8 mcg		
9.5 mcg	2.25 mcg	¼ grain (15 mg)	25 mcg (0.025mg)
19 mcg	4.5 mcg	½ grain (30 mg)	50 mcg (0.05mg)
28.5 mcg	6.75 mcg		75 mcg (0.075mg)
33.44 mcg	7.92 mcg		88 mcg (0.088mg)
38 mcg	9 mcg	1 grain (60 mg)	100 mcg (0.1mg)
42.56 mcg	10.08 mcg		112 mcg (0.112mg)
47.5 mcg	11.25 mcg		125 mcg (0.125mg)
52.06 mcg	12.33 mcg		137 mcg (0.137mg)
57 mcg	13.5 mcg	1 ½ grain (90 mg)	150 mcg (0.15mg)
66.5 mcg	15.75 mcg		175 mcg (0.175mg)
76 mcg	18 mcg	2 grain (120 mg)	200 mcg (0.2mg)
85.5 mcg	20.25 mcg		
95 mcg	22.5 mcg		
114 mcg	27 mcg	3 grain (180 mg)	300 mcg (0.3mg)
152 mcg	36 mcg	4 grain (240 mg)	
190 mcg	45 mcg	5 grain (300 mg)	

Supplement 1. Example of compounding pharmacy conversion chart to convert T4 to T4/T3
